# Supplementary material for: Content-rich biological network constructed by mining PubMed abstracts
Source: BMC Bioinformatics. 2004 Oct 8;5:147. doi: 10.1186/1471-2105-5-147 (PMC528731; doi:10.1186/1471-2105-5-147)
Supplement: Additional File 2 — The original results of the above study (non-essential files are deleted to keep the file size under the limit set by BMC bioinformatics). [file 1471-2105-5-147-S2.bz2 › chilibotAdditionalFile2/dip20/722ID2167176E18062/html/SECE_SECY.html]

 


 **SECE** and **SECY** 
  
Found 94 abstracts in PubMed, retrieved 20.  
 

 What does Google say? 
 PDF only 
| .edu only 

---

**Interactive relationship** (e.g. stimulation, inhibition, etc)

**Neutral relationship**- Although the importance of interactions involving both the cytosolic and transmembrane regions of  **SecY**  and  **SecE**  has been documented, no information has been available for the physical contact sites of these translocase subunits in their cytosolic domains.  Ref: 12809500 Biochemistry, 2003
- Both genetic and biochemical data indicate interactions of several transmembrane segments TMSs of  **SecY**  with  **SecE** .  Ref: 11445571 J Biol Chem, 2001
- Mapping the sites of interaction between  **SecY**  and  **SecE**  by cysteine scanning mutagenesis.  Ref: 11445571 J Biol Chem, 2001
- Thus, CBD cellulose interactions can be employed as a salt insensitive affinity purification system for the capture of complexes containing the Haloferax volcanii translocation apparatus components  **SecY**  and  **SecE** .  Ref: 12579382 Extremophiles, 2003
- By exploiting the salt insensitive interaction of the cellulose binding domain CBD of the Clostridium thermocellum cellulosome with cellulose, purification of CBD fused versions of  **SecY**  and  **SecE** , components of the translocation apparatus of the halophilic archaeon Haloferax volcanii, was undertaken.  Ref: 12579382 Extremophiles, 2003
- We suggest that the cytosolic and intramembrane interactions bring these two subunits together, forming a functionally crucial SecYE interface involving the  **SecY**  C5 region and the conserved cytosolic segment of  **SecE** .  Ref: 12809500 Biochemistry, 2003
- Deletion of the N terminal region of  **SecE**  also rendered the  **SecE**   **SecY**  SecG complex unstable.  Ref: 11129584 Biosci Biotechnol Biochem, 2000
- However, anti HA immunoprecipitates contain neither untagged  **SecE**  nor  **SecY**  cross linked to  **SecE** .  Ref: 10944122 EMBO J, 2000
- ii Cross linking of membranes containing unmodified  **SecE**  and hemagglutinin tagged  **SecE**   **SecE**  HA reveals cross links between  **SecY**  and  **SecE**  and between  **SecY**  and  **SecE**  HA.  Ref: 10944122 EMBO J, 2000
- Remarkably, this model reveals that TMS 3 of  **SecE**  is strongly tilted relative to  **SecY** .  Ref: 12138117 J Biol Chem, 2002
- In this report we show that cpSecE in cells depleted for bacterial  **SecE**  i supports growth, ii stabilizes, just like bacterial  **SecE** , the Sec translocase core component  **SecY** , and iii supports Sec dependent protein translocation.  Ref: 11389897 FEBS Lett, 2001
- These cross linkages were abolished by the Gly240 mutation in the  **SecY**  C4 region as well as by prlG alterations in  **SecE**  transmembrane segment 3, known to compromise  **SecY**   **SecE**  interaction.  Ref: 12809500 Biochemistry, 2003
- A  **SecE**  mutation that modulates  **SecY**   **SecE**  translocase assembly, identified as a specific suppressor of  **SecY**  defects.  Ref: 12533470 J Bacteriol, 2003

**Non-interactive relationship** (e.g. studied together, co-existance, homology, etc.)

- Overproduction of  **SecE** , but not of  **SecY** , alleviated the dominant negative effects.  Ref: 12655407 Mol Genet Genomics, 2003
- In the bacterial cytoplasmic membrane, the channel is formed by oligomers of a heterotrimeric membrane protein complex consisting of subunits  **SecY** ,  **SecE**  and SecG.  Ref: 12167867 Nature, 2002
- Evidence indicates that the Ser105 to Pro alteration in the C terminal transmembrane segment of  **SecE**  weakens  **SecY**   **SecE**  association.  Ref: 12533470 J Bacteriol, 2003
- By means of cysteine scanning mutagenesis, we have identified intermolecular sites of contact between TMS7 of  **SecY**  and TMS3 of  **SecE** .  Ref: 11445571 J Biol Chem, 2001
- The cross linking of  **SecY**  to  **SecE**  demonstrates that these subunits are present in a one to one stoichiometry within the SecYEG complex.  Ref: 11445571 J Biol Chem, 2001
- Whereas homologues of Sec61alpha  **SecY**  and Sec61gamma  **SecE**  exist in archaea, identification of the third component Sec61beta or SecG has remained elusive.  Ref: 11943537 Trends Biochem Sci, 2002
- i Formaldehyde cross linking of translocase reveals cross links between  **SecY** ,  **SecE**  and SecG, but not higher order oligomers.  Ref: 10944122 EMBO J, 2000
- **SecY** ,  **SecE**  and SecG form a heterotrimer, which functions as a protein translocation channel in Escherichia coli.  Ref: 12655407 Mol Genet Genomics, 2003
- We now demonstrate that  **SecE**  also contacts TMS 10 of  **SecY** .  Ref: 12138117 J Biol Chem, 2002
- **SecY**  and  **SecE**  are the two principal translocase subunits that create a channel like pathway for the transit of preprotein across the bacterial cytoplasmic membrane.  Ref: 12533543 J Biol Chem, 2003
- An artificially constructed TM9 PhoA protein, derived from  **SecY** , as well as the  **SecE**  protein, were sensitized to FtsH by addition of extra amino acid sequences to their N terminal cytosolic tails.  Ref: 11256624 EMBO Rep, 2000
- YidC could not be integrated in the absence of  **SecY** ,  **SecE** , or SecG, suggesting that YidC, in contrast to its mitochondrial orthologue Oxa1p, cannot engage a SecYEG independent protein conducting channel.  Ref: 11777926 J Biol Chem, 2002
- Identification of a  **SecY**   **SecE**  Cytosolic Interface.  Ref: 12809500 Biochemistry, 2003
- Cysteines introduced into  **SecY**  residue 244 in the fourth cytosolic domain C4 as well as into residues 354 356 and 362 in the C5 domain could be cross linked with natural or engineered residues at positions 79 and 81 in the central part of the cytosolic loop of  **SecE** .  Ref: 12809500 Biochemistry, 2003
- Previous cysteine scanning studies showed that the transmembrane segment TMS 3 of  **SecE**  contacts TMS 2 and 7 of  **SecY** , and TMS 3 of another  **SecE** .  Ref: 12138117 J Biol Chem, 2002
- Isolation of fusion proteins containing  **SecY**  and  **SecE** , components of the protein translocation complex from the halophilic archaeon Haloferax volcanii.  Ref: 12579382 Extremophiles, 2003
- These prokaryotic eukaryotic proteins include 1  **SecY**  Sec61alpha, 2  **SecE**  Sec61gamma, 3 SecG Sec61beta, 4 Ffh SRP54 and 5 FtsY SRP receptor subunit alpha.  Ref: 12507766 Biochim Biophys Acta, 2003
- The majority are exported through pores formed by  **SecY**  and  **SecE** .  Ref: 11051763 Prog Nucleic Acid Res Mol Biol, 2001
- We now carried out site specific cross linking experiments to identify  **SecY**  and  **SecE**  regions that are physically close.  Ref: 12809500 Biochemistry, 2003
- SecYEG, a complex of integral membrane proteins  **SecY** ,  **SecE** , and SecG in the cytoplasmic membrane, constitutes a pathway for polypeptide movement.  Ref: 12812064 Yi Chuan Xue Bao, 2003
- Although cross linking reversibly inactivates translocation, the contact between TMS7 of  **SecY**  and TMS3 of  **SecE**  remains unaltered upon insertion of the preprotein into the translocation channel.  Ref: 11445571 J Biol Chem, 2001
- Escherichia coli preprotein translocase comprises a membrane embedded trimeric complex of  **SecY** ,  **SecE**  and SecG.  Ref: 11867527 EMBO J, 2002
- While  **SecE**  and  **SecY**  are generally accepted as essential components of translocase, the role of SecG is more ambiguous.  Ref: 10732681 Mol Gen Genet, 2000
- **SecY** ,  **SecE**  and SecG form the membrane embedded core complex of the Escherichia coli protein export apparatus.  Ref: 10732681 Mol Gen Genet, 2000
- Integral membrane components  **SecY** ,  **SecE** , and SecG of protein translocase form a complex in the Escherichia coli plasma membrane.  Ref: 12809499 Biochemistry, 2003
- Following transformation of Haloferax volcanii cells with CBD  **SecY**  or  **SecE**  encoding plasmids, cellulose based purification led to the capture of stably expressed, membrane bound 68 and 25 kDa proteins, respectively.  Ref: 12579382 Extremophiles, 2003
- The integral membrane proteins  **SecY**  and  **SecE**  are conserved core subunits of the translocase.  Ref: 12138117 J Biol Chem, 2002
